# Comprehensive insights into the disease burden linked to air pollution exposure across U.S. states: findings from the 2021 Global Burden of Disease Study

**DOI:** 10.3389/fpubh.2025.1636544

**Published:** 2025-07-23

**Authors:** Kaibin Lin, Junxian Quan, Jiafen Liao, Yiyue Chen, Bing Zhou, Xi Xu

**Affiliations:** ^1^School of Computer Science, Hunan First Normal University, Changsha, China; ^2^Department of Rheumatology and Immunology, The Second Xiangya Hospital of Central South University, Changsha, China; ^3^Clinical Medical Research Center for Systemic Autoimmune Diseases in Hunan Province, Changsha, China; ^4^Clinical Nursing Teaching and Research Section, The Second Xiangya Hospital of Central South University, Changsha, China; ^5^School of Computer Science, Hunan University of Technology, ZhuZhou, China

**Keywords:** GBD 2021, air pollution, disease burden, PM_2_.5, ozone

## Abstract

**Background:**

Ambient air pollution persists as a critical global health threat, ranking fourth among risk factors for premature mortality. Despite decades of air quality improvements in the U.S. through regulatory measures, persistent health impacts remain, driven primarily by particulate matter (PM_2_.5) and ozone.

**Objective:**

This study aimed to quantify long-term trends (1990–2021) in air pollution-attributable disease burdens across U.S. states, evaluate the effectiveness of existing policies, and identify priorities for future public health strategies to address persistent and emerging risks.

**Methods:**

Using the Global Burden of Disease (GBD) 2021 dataset, we analyzed age-standardized mortality, disability-adjusted life years (DALYs), years of life lost (YLLs), and years lived with disability (YLDs) attributable to PM_2_.5, ozone, and household air pollution. Disease burdens were assessed for chronic obstructive pulmonary disease (COPD), diabetes mellitus, ischemic heart disease (IHD), lower respiratory infections, stroke, and lung cancer. Data were standardized to the GBD reference population for comparability over time.

**Results:**

From 1990 to 2021, PM_2_.5-attributable mortality declined by 80.5%, with IHD deaths falling by 70.6% (79,684–23,433 deaths) and associated DALYs by 71.2%. However, diabetes-related YLDs surged 97.4% nationally, reflecting interactions with obesity and lifestyle factors. Formerly high-pollution states (e.g., Indiana, Tennessee) achieved substantial (30%−40%) reductions in PM_2_.5-linked DALYs for IHD and COPD, while California saw a 12.3% rise in diabetes DALYs. In 2021, residual burdens disproportionately affected older adults and males, with IHD mortality rates 1.8 times higher in men. Ozone-related COPD deaths showed minimal decline despite falling ozone levels.

## 1 Introduction

Exposure to ambient air pollution increases deaths and morbidity, reducing life expectancy (1, 2). Ambient air pollution is the fourth-leading global risk factor for premature death, following hypertension, smoking, and poor diet (3, 4). It adversely affects most organ systems and is associated with various debilitating diseases, including asthma, cardiovascular disease, chronic obstructive pulmonary disease (COPD), pneumonia, stroke, diabetes, lung cancer, and dementia (5).

Improved air quality can yield relatively rapid and sustained health benefits (6). In recent decades, air pollution levels in many high-income countries have significantly declined, primarily due to effective regulatory actions and reduced emissions from major sources such as transportation and power generation (7).

However, recent evidence indicates that health effects persist even at pollution levels below current ambient air quality standards (8–10). The Health Effects Institute (HEI) conducted a comprehensive research program evaluating the health impacts of long-term exposure to low-level air pollution, focusing on all-cause and cause-specific deaths and morbidity (11). Low-level air pollution was defined as concentrations below the current annual average air quality standards in the US, Canada, and Europe at the time of the study (7). The annual National Ambient Air Quality Standard for PM_2_.5 in the U.S. was recently lowered from 12 to 9 μg/m3 by the Environmental Protection Agency (EPA) in early 2024 (12).

Despite substantial efforts and investments by the US to improve air quality since the 1970s (6), ambient air pollution continues to threaten public health (13). This study, therefore, aims to provide a comprehensive evaluation of the disease burden trends attributable to air pollution across the U.S. from 1990 to 2021. By focusing on six major pollution-related diseases [COPD, diabetes, ischemic heart disease (IHD), lower respiratory infections, tracheal, bronchial, and lung cancers, and stroke], we not only quantify these long-term changes but also qualitatively explore their connections to the broader context of U.S. environmental policy, public health advancements, and shifting population health dynamics. Our findings are intended to highlight persistent challenges and inform future public health strategies.

## 2 Methods

### 2.1 Data source and study design

This study is a secondary analysis of publicly available data from the GBD 2021 study. Our work involved extracting, processing, and visualizing state-level data for the United States from 1990 to 2021 to analyze trends and patterns in air pollution-attributable disease burden. The core estimation of exposure and attributable burden was performed by the GBD collaboration, as described below. All analyses were based on publicly available data, thus requiring no further ethical approval.

### 2.2 GBD framework for estimating air pollution-attributable burden

The GBD 2021 methodology for quantifying the disease burden attributable to air pollution involves a comprehensive five-step comparative risk assessment framework (3, 14).

Exposure estimation: Ambient PM_2_.5 and ozone concentrations were estimated at a high-resolution grid (e.g., 1 × 1 km) by integrating satellite data, ground-level monitoring station measurements, and chemical transport models (15). To derive the summary exposure levels for national and state populations used in health impact assessment, GBD calculates population-weighted annual average concentrations. This method assigns greater weight to the estimated concentrations in more densely populated grid cells, thus better representing the average exposure level experienced by the population. Household air pollution exposure was estimated based on the proportion of households using solid fuels for cooking, derived from household surveys and census data (3).

Identification of risk-outcome pairs: GBD identifies causal links between pollutants and specific diseases based on extensive systematic reviews of epidemiological literature. For this study, the relevant pairs include PM_2_.5 with six outcomes (IHD, stroke, COPD, lung cancer, lower respiratory infections, and diabetes) and ozone with one outcome (COPD) (3).

Estimation of relative risks: Integrated Exposure-Response (IER) functions were used to model the relationship between exposure levels and the risk of disease. These non-linear functions are derived from pooling data from diverse cohort studies and define the increase in disease risk across the full global range of pollutant concentrations (3).

Determination of theoretical minimum risk exposure level (TMREL): GBD defines a TMREL, which represents the exposure level associated with the minimum risk of adverse health outcomes, based on the lowest levels observed in epidemiological studies. For PM_2_.5, the TMREL is defined as a range of 2.4–5.9 μg/m3 (3).

Calculation of population attributable fraction (PAF): for each pollutant-disease pair, state, year, age, and sex, the PAF was calculated. The PAF represents the proportion of disease cases that would be avoided if exposure were reduced to the TMREL. It is calculated using the relative risks derived from the IER functions and the distribution of exposure levels in the population (3, 14).

The final attributable burden [e.g., deaths, disability-adjusted life years (DALYs)] was then computed by multiplying the total burden of a specific disease (e.g., total IHD deaths) by its corresponding PAF. All estimates are produced with 95% uncertainty intervals (UIs) derived from 1,000 draws of the calculation process.

### 2.3 Health metrics and age standardization

We analyzed four key metrics from the GBD 2021 dataset: DALYs, years of life lost (YLLs) due to premature mortality, and years lived with disability (YLDs).

YLLs were calculated by multiplying the number of deaths in each age group by the standard life expectancy at that age from the GBD reference life table. YLDs were calculated by multiplying the prevalence of a disease by its disability weight, which reflects the severity of the condition. DALYs are the sum of YLLs and YLDs, representing the total years of healthy life lost to a specific cause.

To ensure comparability across different populations and time periods, all rates were age-standardized. This statistical process adjusts for differences in population age structures by applying the observed age-specific rates for each state and year to a standard reference population, namely the GBD 2019 global age-standard population. This removes the confounding effect of a population aging over time, allowing for a clearer assessment of changes in health risks (3, 14).

### 2.4 Statistical analysis

Data processing and statistical analyses were performed using Python (version 3.10.9) and R (version 4.4.2). Data cleaning and integration were conducted with Python 3.10.9 using pandas, version 2.2.3, while R packages employed included plotly (version 4.10.4), and dplyr (version 1.1.4) for data organization and analysis. For visualization, temporal trend line graphs and geographical distribution maps were generated using Matplotlib (version 3.5.3) in Python and ggplot2 (version 3.5.1) in R, respectively. Additionally, the R package plotrix (version 3.8-4) was used for data visualization enhancements, and RColorBrewer (version 1.1-3) facilitated the creation of color gradients. All analyses were based on publicly available data from GBD 2021, thus requiring no further ethical approval.

## 3 Results

### 3.1 Trends in disease burden attributable to air pollution exposure in the US (1990–2021)

**Figure 1:**
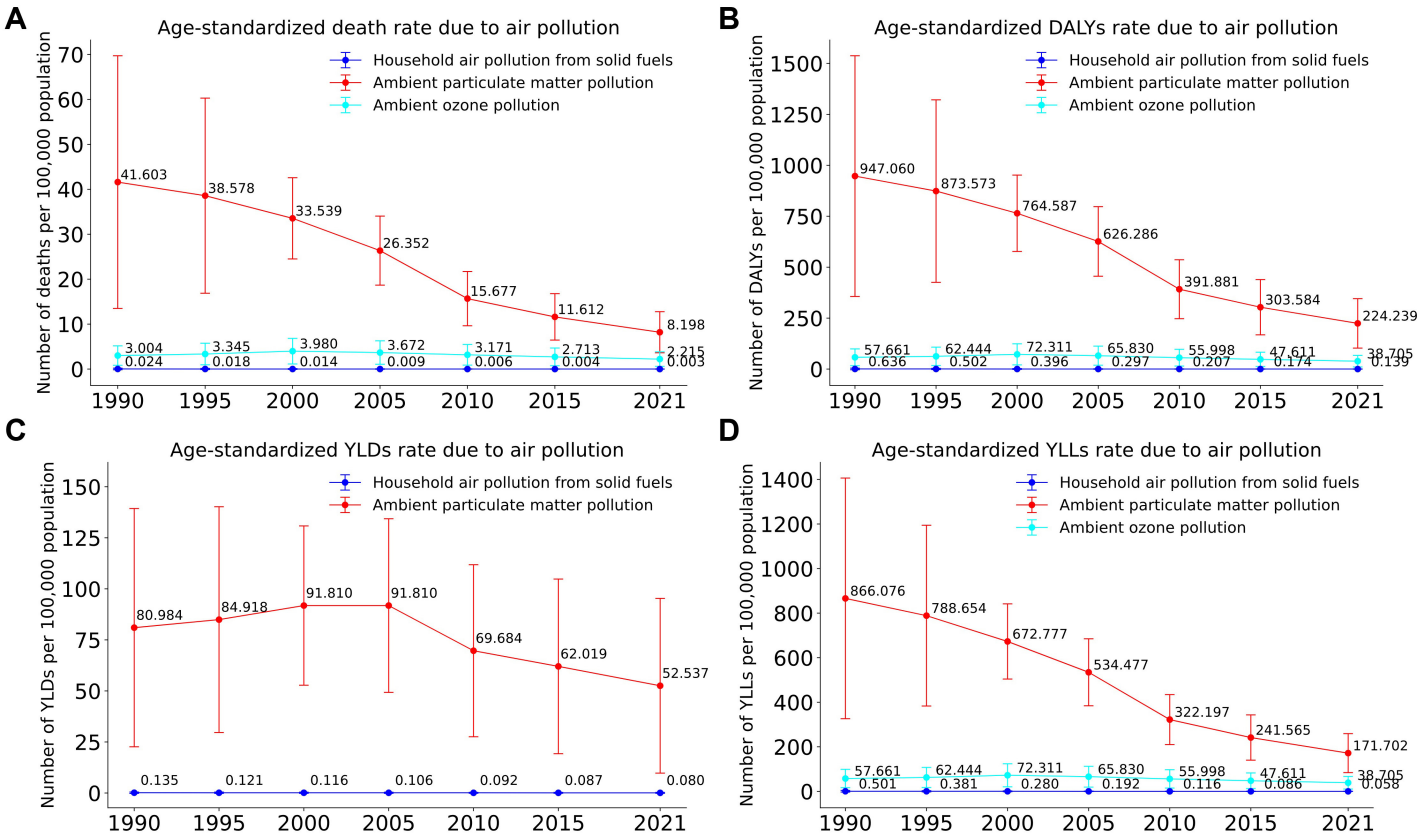
Trends in age-standardized disease burden attributable to three major air pollution sources in the US, 1990–2021. **(A)** Age-standardized death rate. **(B)** Age-standardized DALYs rate. **(C)** Age-standardized YLDs rate. **(D)** Age-standardized YLLs rate.

From 1990 to 2021, the overall disease burden attributable to air pollution exposure in the US showed a declining trend (Figure 1), indicating significant progress in reducing air pollution exposure and mitigating its associated health impacts. Among the studied pollution sources, ambient particulate matter pollution (PM_2_.5; red line) remained the leading contributor to adverse health outcomes throughout the entire period. Although its burden was substantially reduced, ambient particulate matter pollution remained a greater contributor to health loss than ambient ozone pollution (cyan line) throughout the entire study period. Household air pollution from solid fuels (blue line) was associated with the lowest burden, which continued to decline throughout the period.

The age-standardized death rate attributable to ambient particulate matter pollution decreased significantly, from ~41.6 (95% UI: 18.1–69.7) per 100,000 individuals in 1990 to about 8.2 (95% UI: 4.1–12.8) per 100,000 in 2021. Similarly, ozone-related deaths declined from roughly 3.0 (95% UI: 0.7–5.2) per 100,000 to around 2.2 (95% UI: 0.5–3.8) per 100,000, while deaths linked to household air pollution remained consistently low, dropping from 0.024 (95% UI: 0.000–0.198) per 100,000 to 0.003 (95% UI: 0.000–0.016) per 100,000. Trends for DALYs (Figure 1B) and YLLs (Figure 1D) mirrored this pattern, with the DALY rate from particulate matter declining from 947.1 (95% UI: 433.8–11,537.8) in 1990 to 224.2 (95% UI: 118.1–345.5) in 2021, consistently dominating these metrics but showing considerable declines overall. In contrast, Figure 1C (YLDs) indicated that particulate matter continued to have the highest share of YLDs, although this began to gradually decrease after 2000.

Despite these improvements, the persistent health burden of ambient particulate matter and ozone pollution in 2021 remains a critical concern, highlighting the ongoing need for targeted pollution control measures and public health interventions.

### 3.2 Trends in disease burden from ambient air pollution by disease category in the US

To evaluate the long-term health impacts of ambient air pollution in the US, we analyzed disease burden data from 1990 to 2021 extracted from the GBD 2021 database, focusing on six major diseases: IHD, diabetes mellitus, COPD, lower respiratory infections, stroke, and tracheal, bronchial, and lung cancers.

**Figure 2:**
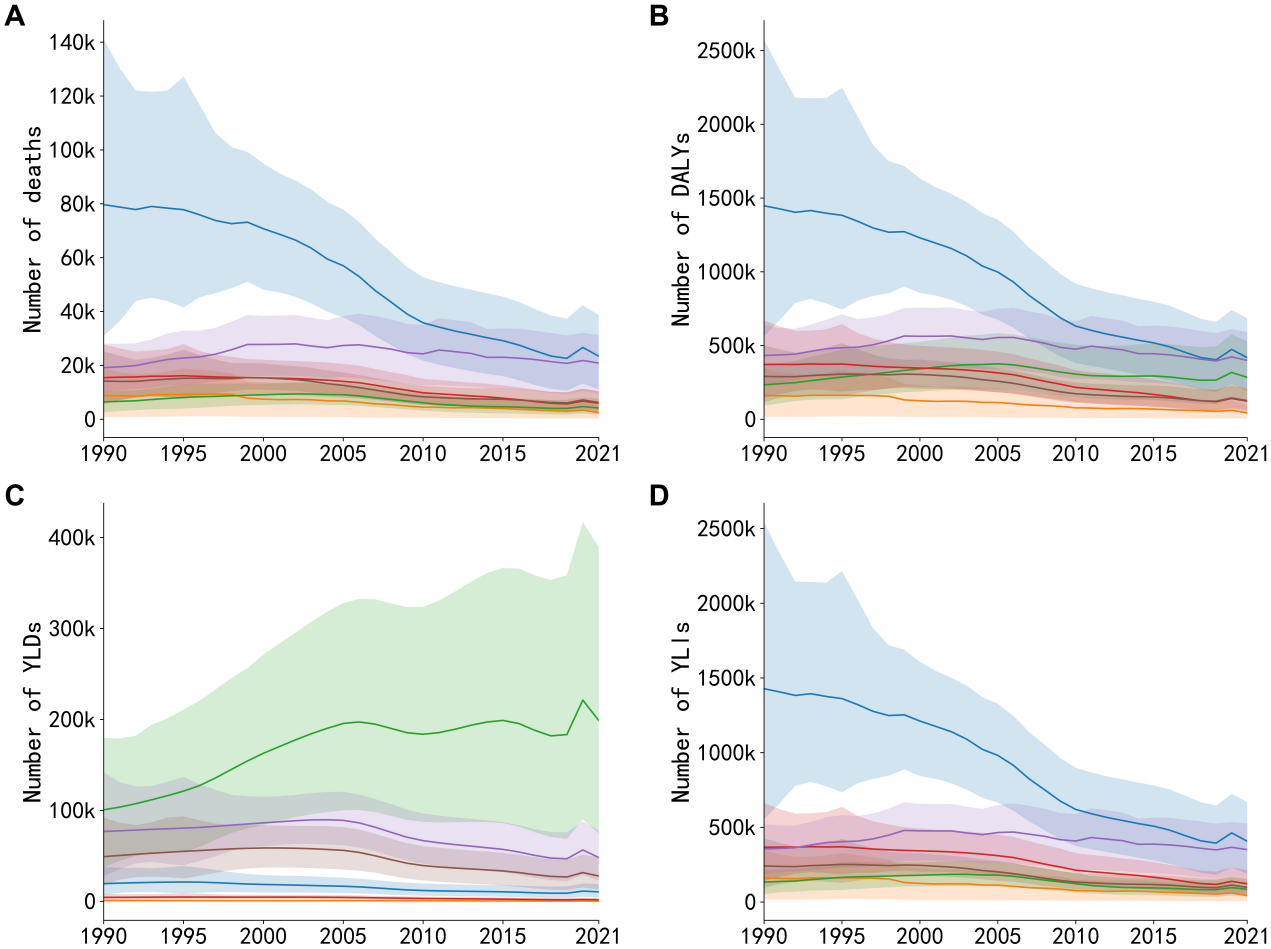
Trends in deaths, DALYs, YLDs, and YLLs attributable to six major diseases caused by air pollution exposure in the US, 1990–2021. **(A)** Annual number of deaths. **(B)** Annual number of DALYs. **(C)** Annual number of YLDs. **(D)** Annual number of YLLs.

In Figure 2A, ischemic heart disease accounted for the largest share of total deaths, declining substantially from 79,684 (95% UI: 79,684 [554,122–2,541,881]) deaths in 1990 to 23,433 (95% UI: 11,831–38,093) in 2021—a reduction of ~70.6%. Although the number of deaths from diabetes, stroke, COPD, lower respiratory infections, and lung cancers also decreased, the declines were relatively modest in comparison. Trends in DALYs (Figure 2B) and YLLs (Figure 2D) showed similar patterns, highlighting significant reductions in the overall burden associated with ischemic heart disease. Specifically, DALYs attributable to ischemic heart disease decreased markedly from 1,447,730 (95% UI: 554,122–2,541,881) to 417,662 (95% UI: 188,310–668,093), representing a reduction of 71.2%. Similarly, YLLs declined from 1,428,000 (95% UI: 554,122–2,541,881) in 1990 to 407,182 (95% UI: 188,310–668,093) in 2021, reflecting a 72.5% decrease.

In contrast, as shown in Figure 2C, YLDs from diabetes notably increased from 100,690 (95% UI: 37,662–198,797) in 1990 to 198,797 (95% UI: 97,848–344,291) in 2021, a growth rate of 97.4%, while other diseases exhibited smaller or declining trends. The YLD burden for ischemic heart disease remained relatively low compared to deaths and YLLs, suggesting its primary burden is concentrated in premature deaths rather than chronic disability. These results indicate that among major health risks related to air pollution, premature deaths from cardiovascular disease has significantly decreased; however, metabolic diseases such as diabetes remain a critical issue. Thus, further attention and interventions addressing both environmental and lifestyle risk factors are required.

### 3.3 Regional variations and trends in PM_2_.5 exposure and DALYs for major diseases across US states

**Figure 3:**
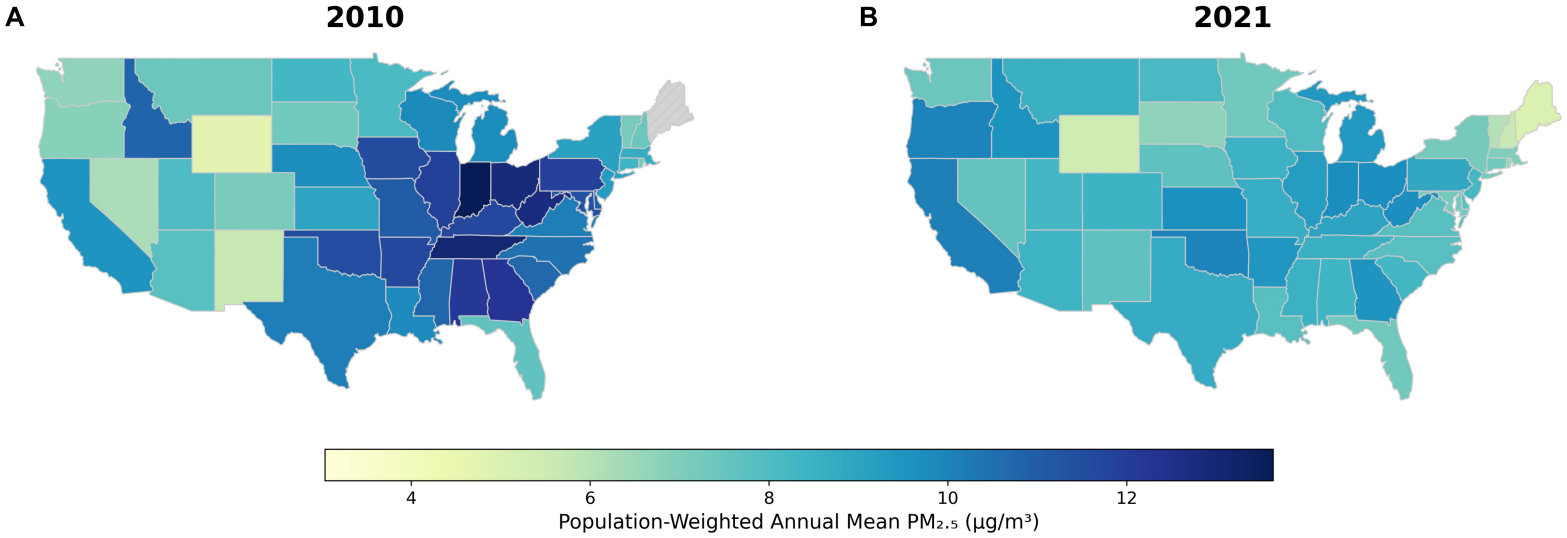
Spatiotemporal changes in PM_2_.5 concentrations and DALYs attributable to major air pollution-related diseases across US states, 2010–2021. **(A)** Concentrations in 2010. **(B)** Concentrations in 2021.

To clearly illustrate recent trends in the health burden attributable to ambient air pollution, we compared state-level data between 2010 and 2021. A significant nationwide decline in ambient PM_2_.5 concentrations occurred during this period. In 2010, the highest concentrations were observed in the East-Central and parts of the Southern U.S., with several states exceeding 12 μg/m3. By 2021, PM_2_.5 levels had decreased across the country, with the most substantial reductions seen in these same historically high-pollution regions (Figure 3).

**Figure 4:**
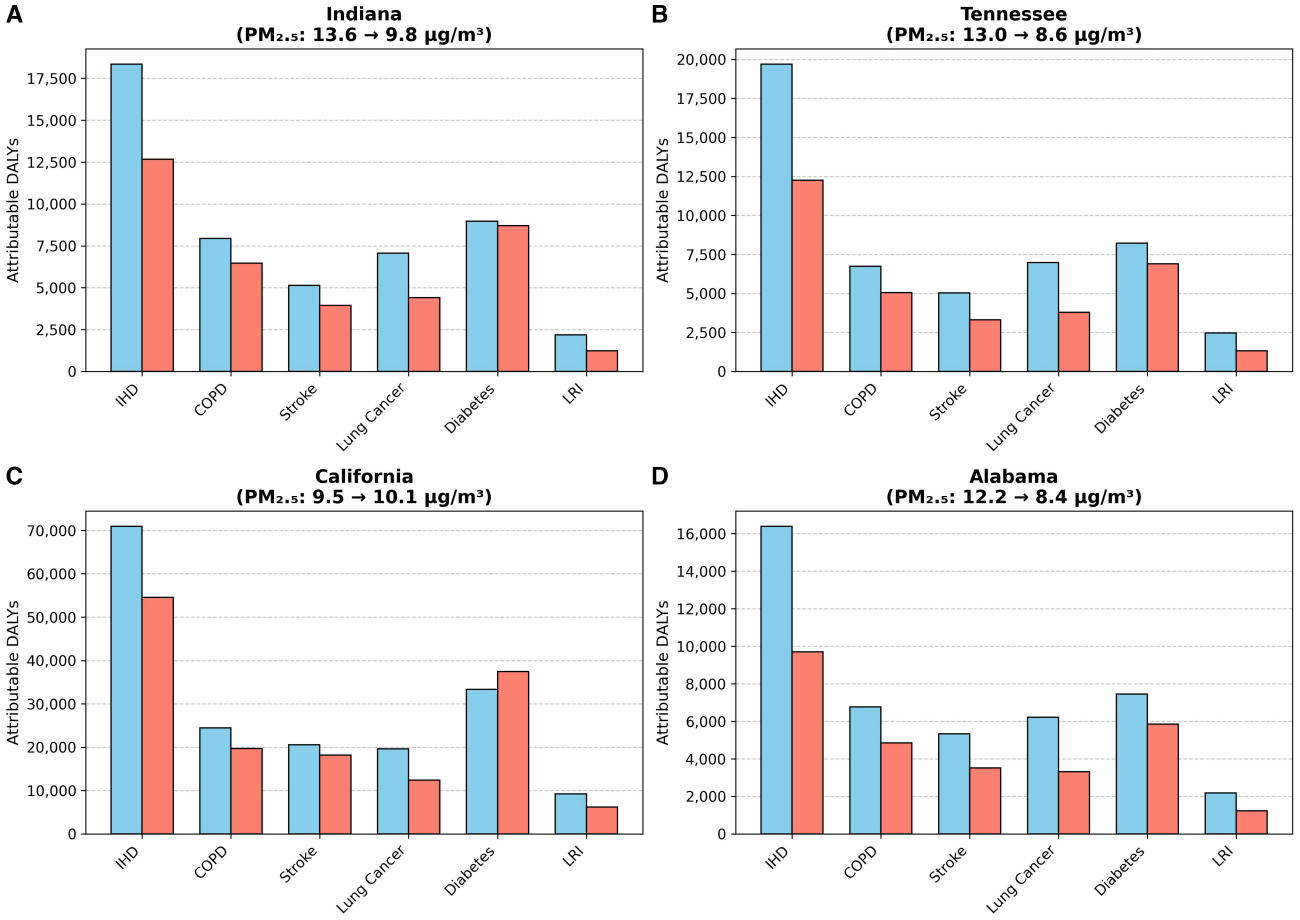
Changes in PM_2_.5-attributable DALYs for key states (2010 vs. 2021). **(A)** Indiana. **(B)** Tennessee. **(C)** California. **(D)** Alabama.

The direct public health benefits of these air quality improvements are evident when examining the changes in attributable DALYs for key states (Figure 4). For instance, Indiana, which saw its average annual PM_2_.5 concentration fall from 13.6 to 9.8 μg/m3, experienced a corresponding 31% reduction in DALYs from IHD and a 19% reduction from COPD. A similar success story is seen in Tennessee, where PM_2_.5 levels dropped by 34% (from 13.0 to 8.6 μg/m3), accompanied by a nearly 38% decrease in IHD-attributable DALYs and significant declines for COPD and lung cancer. In Alabama, a 31% reduction in PM_2_.5 was linked to a 28% drop in COPD-related DALYs and a nearly 47% decrease in DALYs from lung cancer. Detailed data for all states and diseases can be found in Supplementary Table S1.

However, the trend for diabetes-attributable DALYs diverged notably from this pattern. Notably, in California, where PM_2_.5 levels slightly increased from 9.5 to 10.1 μg/m3, diabetes-related DALYs rose by 12.3% (Figure 4). This finding underscores the complex nature of metabolic diseases, suggesting that their burden is influenced by powerful co-factors like obesity and lifestyle, and that reductions in air pollution alone may not be sufficient to reverse their growing impact.

### 3.4 Age and gender disparities in six diseases attributable to air pollution

**Figure 5:**
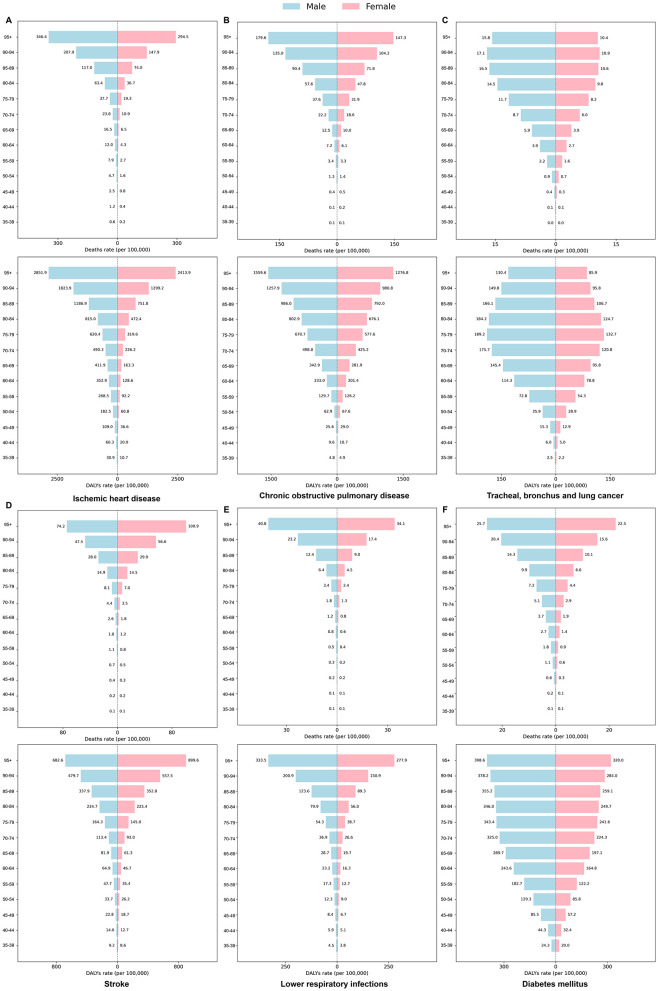
Age- and sex-specific differences in the burden of six diseases attributable to air pollution in the US 2021. The panels show age-specific death and DALYs rates for **(A)** Ischemic heart disease, **(B)** Chronic obstructive pulmonary disease, **(C)** Tracheal, bronchus, and lung cancer, **(D)** Stroke, **(E)** Lower respiratory infections, and **(F)** Diabetes mellitus.

**Figure 6:**
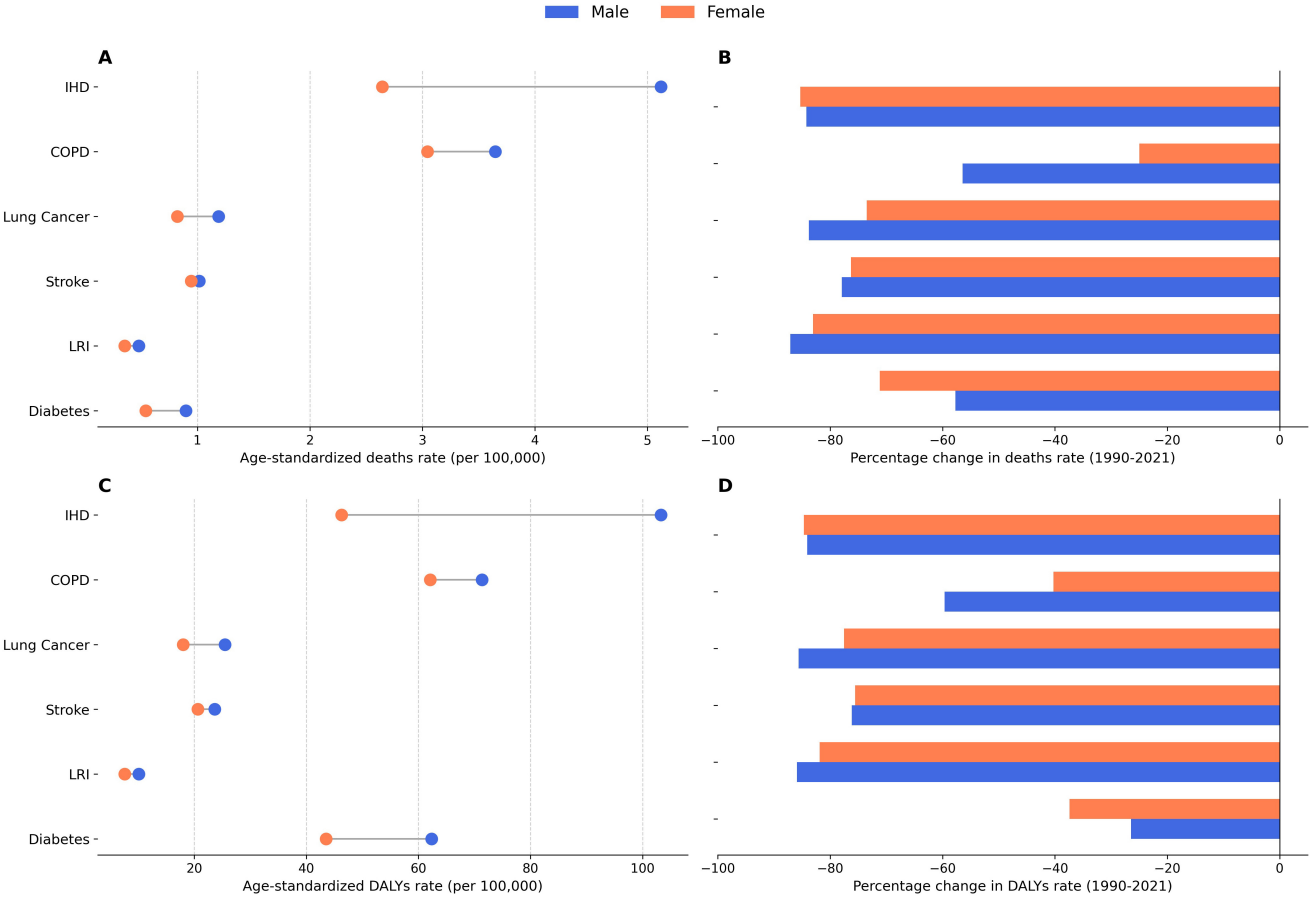
Age-standardized burden of six diseases in 2021 and percentage changes from 1990 to 2021. **(A)** Age-standardized death rates by sex, 2021. **(B)** Percentage change in death rates, 1990–2021. **(C)** Age-standardized DALYs rates by sex, 2021. **(D)** Percentage change in DALYs rates, 1990–2021.

To investigate age- and gender-related differences in the burden of six diseases associated with air pollution, we examined age- and sex-specific data on deaths and DALYs (Figure 5), as well as age-standardized indicators and percentage changes from 1990 to 2021 (Figure 6).

Figure 5 reveals a marked rise in both deaths and DALYs across all six diseases with increasing age, and consistently higher rates among men, particularly in IHD, COPD, and lung cancer. For instance, in IHD, deaths among men aged 95 years or older reached 346.42 per 100,000, compared to 294.45 per 100,000 among women. Notably, stroke deaths in women aged 90 and older exceeded that of men (100.91 vs. 74.23 per 100,000). Among adults 85 years and older, the death rate for lower respiratory infections substantially increased, with the male death rate climbing from 6.36 per 100,000 among those aged 80–84 to 40.75 per 100,000 for those 95 years and above.

Figure 5 illustrates long-term trends between 1990 and 2021. Ischemic heart disease had the highest age-standardized death rate in both men and women (4.5 per 100,000 vs. 3.2 per 100,000, respectively) and also imposed the greatest DALY burden (92.4 vs. 66.1 per 100,000). Meanwhile, COPD demonstrated the largest reduction in DALY rates, with declines of 59.6% in men and 40.2% in women, and IHD deaths decreased by 56.4% (men) and 24.9% (women). Diabetes DALY rates showed comparatively smaller decreases (37.4% in women and 28.1% in men). Lung cancer exhibited the least improvement, particularly among women. Taken together, Figures 5, 6 suggest a downward trend in disease burden over the past 32 years, although persistent age and sex disparities underscore the need for ongoing air quality improvements, early screening, and targeted public health measures to further mitigate the long-term health impacts of air pollution.

An important nuance is observed in the stroke burden. While the overall age-standardized deaths and DALYs rate for stroke were higher in men (Figures 6A, C), the age-specific analysis reveals a crossover pattern, with women exhibiting markedly higher rates in the oldest age groups (e.g., 90+ years; Figure 5). This highlights that summary metrics like age-standardized rates, while useful for overall comparison, can mask critical age-specific vulnerabilities.

### 3.5 Trends in deaths attributable to PM_2_.5 and ozone exposure (2010–2020)

**Figure 7:**
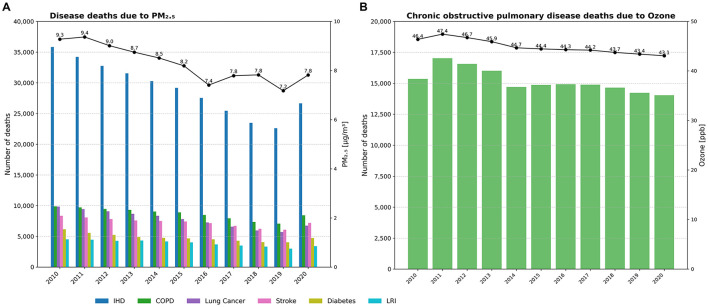
Annual trends in deaths from six diseases attributable to PM_2_.5 and ozone exposure in the US, 2010–2020. **(A)** Deaths from six diseases attributable to PM_2_.5. **(B)** COPD deaths attributable to ozone.

Over the last decade of the study period, U.S. air pollution concentrations generally declined. As shown in Figure 7A, the national population-weighted average PM_2_.5 concentration peaked at 9.4 μg/m3 in 2011, then gradually decreased to its lowest point of 7.2 μg/m3 in 2019, before rising again to 7.8 μg/m3 in 2020. Correspondingly, the total number of deaths from the six diseases linked to PM_2_.5 showed a downward trend, falling from 74,567 in 2010 to 57,147 in 2020.

The grouped bar chart format in Figure 7A clearly illustrates the contribution of each disease. IHD was consistently the largest single contributor to PM_2_.5-attributable deaths, though its annual death toll fell from 35,819 in 2010 to 26,658 in 2020. COPD and tracheal, bronchus, and lung cancers were the next largest contributors, with their death counts showing more modest declines over the decade.

Figure 7B depicts the influence of ambient ozone on COPD. In contrast to the clearer trends for PM_2_.5, national population-weighted average ozone concentrations showed only minor yearly variations between 2010 and 2020, fluctuating between a peak of 47.4 ppb in 2011 and a low of 43.1 ppb in 2020. Consistent with this lack of a clear downward trend in exposure, the number of COPD deaths attributable to ozone also remained relatively stable, fluctuating between ~14,000 and 17,000 annually over the same period.

## 4 Discussion

This study systematically analyzed the effects of air pollution on the major disease burden in the U.S. from 1990 to 2021, leveraging the GBD 2021 dataset. Our findings clearly demonstrate a substantial decline in the overall disease burden attributable to air pollution over the past three decades, a testament to the effectiveness of long-term air quality control measures. However, the analysis also uncovers persistent and emerging challenges, particularly concerning metabolic diseases and ozone-related illnesses, which require targeted public health strategies.

### 4.1 Policy success and drivers of declining PM_2_.5 burden

The most significant finding of our study is the dramatic reduction in PM_2_.5-attributable deaths, primarily driven by a steep decline in ischemic heart disease deaths. This public health success can be largely attributed to the robust implementation of air quality policies in the U.S. The Clean Air Act, enacted in 1970 and subsequently amended, has mandated stringent controls on emissions from both stationary sources (e.g., power plants) and mobile sources (e.g., vehicles) (16). Specific regulations, such as the Acid Rain Program and the Cross-State Air Pollution Rule, effectively curtailed emissions of sulfur dioxide (SO_2_) and nitrogen oxides (NOx), key precursors to secondary PM_2_.5 formation (17). Quantitative studies have confirmed this link; for instance, the EPA's own analysis estimated that for every dollar invested in the Clean Air Act, the return in public health benefits is substantial, with some estimates as high as $30, primarily through reduced premature mortality (18).

These policy drivers were amplified by technological advancements, including the widespread adoption of flue-gas desulfurization in industrial facilities and catalytic converters in vehicles, alongside a national energy transition from coal to natural gas and renewables (6, 19). Furthermore, it is important to acknowledge the synergistic role of a strengthening health system. Over the same period, significant advances in the clinical management of cardiovascular diseases, such as the widespread use of statins, improved hypertension control, and more effective emergency response for acute cardiac events, have likely worked in concert with air quality improvements to lower mortality rates. Our analysis, which extends the timeline of previous work (20, 21) to 2021, confirms that the health benefits of these sustained efforts have continued to accumulate, preventing a substantial number of premature deaths.

### 4.2 Persistent challenges: the paradox of diabetes and ozone burden

In stark contrast to the progress in cardiovascular health, the burden of PM_2_.5-attributable diabetes, particularly in terms of disability (YLDs), has surged. This paradoxical trend highlights the critical interplay between environmental exposures and population-level health dynamics. While PM_2_.5 is a recognized risk factor, likely acting through pathways of systemic inflammation and insulin resistance, the overwhelming driver of this trend is the concurrent obesity epidemic in the U.S. To illustrate, the prevalence of adult obesity in the U.S. soared from ~15% in the early 1990s to over 42% by 2020 (22), dramatically expanding the population susceptible to type 2 diabetes. This has led to a dramatic increase in the underlying prevalence of type 2 diabetes (23–25). Consequently, even with cleaner air, the expanding pool of susceptible individuals results in a higher absolute number of attributable cases, a trend particularly visible in states like California.

Similarly, the deaths burden from ambient ozone, primarily linked to COPD, showed minimal decline. This persistence is multifactorial. Ozone formation is a complex non-linear photochemical process influenced by precursor emissions (NOx and VOCs) and meteorological conditions like temperature and sunlight. Rising global temperatures and more frequent heatwaves associated with climate change may offset some gains from precursor emission controls, creating more favorable conditions for ozone production (21). Furthermore, the health impact of ozone is compounded by a rapidly aging U.S. population, which is inherently more vulnerable to respiratory diseases. The cumulative nature of COPD means that today's mortality rates reflect decades of past exposures, making the burden less responsive to recent fluctuations in pollution levels.

Our trend analysis also reveals a noteworthy pattern in the overall burden metrics: while pollution-attributable YLLs declined steadily from 1990, the corresponding YLDs remained relatively stagnant until the mid-2000s (Figure 1). This divergence likely reflects a complex interplay of competing factors. On one hand, improved air quality and better clinical care for conditions like IHD and COPD reduced premature mortality, thus lowering YLLs. However, this success meant that more patients survived longer while living with chronic disability, which can keep prevalence-based YLDs stable or even increase them. This effect, combined with underlying population growth and aging, likely counteracted some of the expected gains in disability reduction. Furthermore, the rapidly increasing YLD burden from diabetes (Figure 2C) also partially offset the YLD reductions achieved for other major diseases during this period.

### 4.3 Comparison with U.S.-specific burden estimates

It is valuable to contextualize our findings, derived from the globally standardized GBD framework, by comparing them with estimates from U.S.-specific studies. Our analysis, based on the GBD 2021 results, identifies ~57,100 deaths attributable to ambient PM_2_.5 exposure in 2019 across the six specified diseases. This figure is broadly comparable with other recent U.S. estimates, such as the 47,900 (95% CI: 28,100–66,900) premature deaths estimated by (26) for the same year using methodologies preferred by the U.S. EPA (26, 27). Another study by Azimi and Stephens (27), which focused on a framework distinguishing indoor and outdoor sources, estimated ~59,000 deaths in 2014 from ambient PM_2_.5 (28). The differences in these estimates likely stem from several key methodological distinctions. Firstly, the GBD employs global IER functions, which are designed to capture health risks across the full global range of exposures, whereas U.S.-specific studies often utilize concentration-response (C-R) functions derived from North American cohorts. Secondly, variations in the underlying models for estimating PM_2_.5 exposure and the specific study years can contribute to differing results. Finally, the inclusion of diabetes as a PM_2_.5-attributable cause of death in the GBD framework since 2019 may also contribute to different estimates compared to some U.S.-centric models. Importantly, despite these numerical variations, all studies consistently conclude that tens of thousands of deaths in the U.S. remain attributable to PM_2_.5 exposure annually, reinforcing our finding that ambient air pollution continues to be a critical public health threat.

### 4.4 Demographic disparities in air pollution burden

Our study also highlights significant age- and sex-related disparities in the disease burden from air pollution. The results are highly consistent with global research (29), which reports that older adults are more susceptible. Our findings confirm a sharply increasing burden in individuals aged 70 and older. Men also consistently face a higher burden for most diseases, including IHD, COPD, and lung cancer. These sex-based differences may be partly explained by historical and ongoing factors such as higher rates of occupational exposure and smoking among men, a conclusion supported by epidemiological research in other high-income regions (30). These disparities underscore the need for targeted public health interventions aimed at protecting vulnerable populations.

Placing our findings within a global context enhances their relevance and potential for generalization. The substantial decline in the U.S. air pollution burden over three decades, driven by sustained policy and technological shifts, offers a model of success for high-income nations. This trajectory aligns with broader trends observed in other developed economies, where economic growth has eventually been coupled with environmental quality improvements, as explored in studies on OECD countries (31). However, this success starkly contrasts with the situation in many rapidly industrializing nations. For instance, a recent GBD-based study on BRICS countries by (32) highlights a rising or persistently high disease burden from air pollution during a similar period, underscoring the different challenges faced at various stages of economic development (32). Therefore, while the specific policy instruments may differ, the U.S. experience underscores a more universal lesson: long-term, consistently enforced environmental regulations are fundamental to decoupling economic activity from public health harm, a principle applicable to developed and developing countries alike.

### 4.5 Strengths and limitations

A major strength of this study is its use of the comprehensive and standardized GBD 2021 dataset. However, certain limitations must be acknowledged.

First, and most importantly, our study is descriptive in nature. We did not perform a formal regression analysis to quantify the specific contributions of socioeconomic factors, policy interventions, or health system indicators to the observed changes in disease burden. Such a quantitative analysis would be a valuable, though complex, next step for future research. Instead, our study provides a comprehensive overview of the trends, which serves as a crucial foundation for formulating hypotheses for these future analytical studies.

Second, this study relies on modeled estimates from the GBD, which are subject to uncertainty. As shown in Figure 1, the UIs are considerably wide, particularly for earlier years in the study period. This greater uncertainty in the 1990s likely reflects the relative scarcity of ground-level air quality monitoring data and other input data available for that era compared to more recent years. While these estimates represent the most robust data currently available, the UIs underscore the importance of interpreting the trends with an appreciation for the underlying modeling complexities.

Third, our state-level analysis, while useful for policy overview, can mask significant health disparities and exposure heterogeneity at the county or community level.

Finally, this study did not extensively explore the interactions between air pollution and socioeconomic status, which is a critical determinant of health and a vital area for future research.

## 5 Conclusions

This study comprehensively reveals the long-term trends (1990–2021) of six major diseases attributed to air pollution in the US. Our findings confirm that decades of air quality improvement measures have successfully reduced the health burden of ambient air pollution, especially in lowering deaths and DALYs related to ischemic heart disease, chronic obstructive pulmonary disease, and lung cancer. However, the increasing burden of metabolic diseases such as diabetes indicates that future public health interventions must address not only air pollution control but also other critical risk factors, including obesity and insufficient physical activity.

At the same time, this study underscores pronounced age- and sex-related disparities in the disease burden of air pollution, with older adults and men remaining at higher risk. Accordingly, future public health policies should continue striving to further reduce air pollution levels, implement targeted interventions for high-risk older and male populations, and strengthen chronic disease prevention strategies to achieve more substantial public health benefits.

## Data Availability

The raw data supporting the conclusions of this article will be made available by the authors, without undue reservation.
